# C1q/TNF-Related Protein-9 Ameliorates Ox-LDL-Induced Endothelial Dysfunction via PGC-1α/AMPK-Mediated Antioxidant Enzyme Induction

**DOI:** 10.3390/ijms18061097

**Published:** 2017-05-26

**Authors:** Haijian Sun, Xuexue Zhu, Yuetao Zhou, Weiwei Cai, Liying Qiu

**Affiliations:** 1 Department of Basic Medicine, Wuxi School of Medicine, Jiangnan University, Wuxi 214122, China; haijsunjiangnan@jiangnan.edu.cn (H.S.); xxzjn@jiangnan.edu.cn (X.Z.); yuetao.zhou@jiangnan.edu.cn (Y.Z.); vivi_cha@jiangnan.edu.cn (W.C.); 2 Department of Basic Medicine, Wuxi Medical School, Jiangnan University, 1800 Lihu Avenue, Wuxi 214122, China

**Keywords:** endothelial dysfunction, ox-LDL, AMPK, PGC1-α, CTRP9

## Abstract

Oxidized low-density lipoprotein (ox-LDL) accumulation is one of the critical determinants in endothelial dysfunction in many cardiovascular diseases such as atherosclerosis. C1q/TNF-related protein 9 (CTRP9) is identified to be an adipocytokine with cardioprotective properties. However, the potential roles of CTRP9 in endothelial function remain largely elusive. In the present study, the effects of CTRP9 on the proliferation, apoptosis, migration, angiogenesis, nitric oxide (NO) production and oxidative stress in human umbilical vein endothelial cells (HUVECs) exposed to ox-LDL were investigated. We observed that treatment with ox-LDL inhibited the proliferation, migration, angiogenesis and the generation of NO, while stimulated the apoptosis and reactive oxygen species (ROS) production in HUVECs. Incubation of HUVECs with CTRP9 rescued ox-LDL-induced endothelial injury. CTRP9 treatment reversed ox-LDL-evoked decreases in antioxidant enzymes including heme oxygenase-1 (HO-1), nicotinamide adenine dinucleotide phosphate (NAD(P)H) dehydrogenase quinone 1, and glutamate-cysteine ligase (GCL), as well as endothelial nitric oxide synthase (eNOS). Furthermore, CTRP9 induced activation of peroxisome proliferator-activated receptor γ co-activator 1α (PGC1-α) and phosphorylation of adenosine monophosphate-activated protein kinase (AMPK). Of interest, AMPK inhibition or PGC1-α silencing abolished CTRP9-mediated antioxidant enzymes levels, eNOS expressions, and endothelial protective effects. Collectively, we provided the first evidence that CTRP9 attenuated ox-LDL-induced endothelial injury by antioxidant enzyme inductions dependent on PGC-1α/AMPK activation.

## 1. Introduction

Vascular lesion is one of the most important complications in many cardiovascular diseases including atherosclerosis [1]. Oxidized low-density lipoprotein (ox-LDL) is a stress signal contributing to pathogenesis of atherosclerosis [2,3]. The endothelial dysfunction in the vasculature may be an accelerator in the development and progression of atherosclerotic injury [4]. Vascular endothelium is a major contributor in the vascular homeostasis [5]. Endothelial cells are key components in the vascular endothelium, which can produce numerous factors that modulate angiogenesis, inflammation, oxidative stress, nitric oxide (NO) production, vascular tone, and permeability [6]. The proliferation and migration of endothelial cells are essential for angiogenesis, which is a physiological or pathological neovascularization process [7]. The endothelial cell apoptosis is involved in the pathogenesis of atherosclerosis [8]. NO released by vascular endothelial cell is one of the vasoactive substances, which participates in a host of signaling pathways, thereby maintaining normal endothelial function [9]. Besides, NO is demonstrated to protect endothelial cells from pro-inflammatory cytokines, platelet aggregation, and adhesion molecules expressions induced by ox-LDL [10]. The excessive reactive oxygen species (ROS) production in the endothelial cells are requisite step in endothelial dysfunction [11].

C1q-TNF-related protein-9 (CTRP9), the closest adiponectin paralog, functions as a critical regulator in vascular relaxation [12], myocardial injury [13], and vascular smooth muscle cell proliferation [14]. CTRP9 is downregulated and exerts glucose-lowering effects in obese mice [15]. CTRP9 is established to inhibit the macrophage inflammation to increase carotid plaque stability [16]. Moreover, CTRP9 abrogates tumor necrosis factor α-induced endothelial inflammation [17]. These extensive observations suggest that CTRP9 may critically participate in the vascular homeostasis. However, whether and how CTRP9 was involved in endothelial dysfunction in response to ox-LDL remained largely obscure. Therefore, we investigated the potential roles of CTRP9 in ox-LDL-stimulated endothelial injury and the underlying molecular mechanisms. 

## 2. Results

### 2.1. C1q/TNF-Related Protein 9 (CTRP9) Reversed the Proliferation, Migration, Angiogenesis, and Apoptosis in Human Umbilical Vein Endothelial Cells (HUVECs) Response to Oxidized Low-Density Lipoprotein (Ox-LDL)

**Figure 1:**
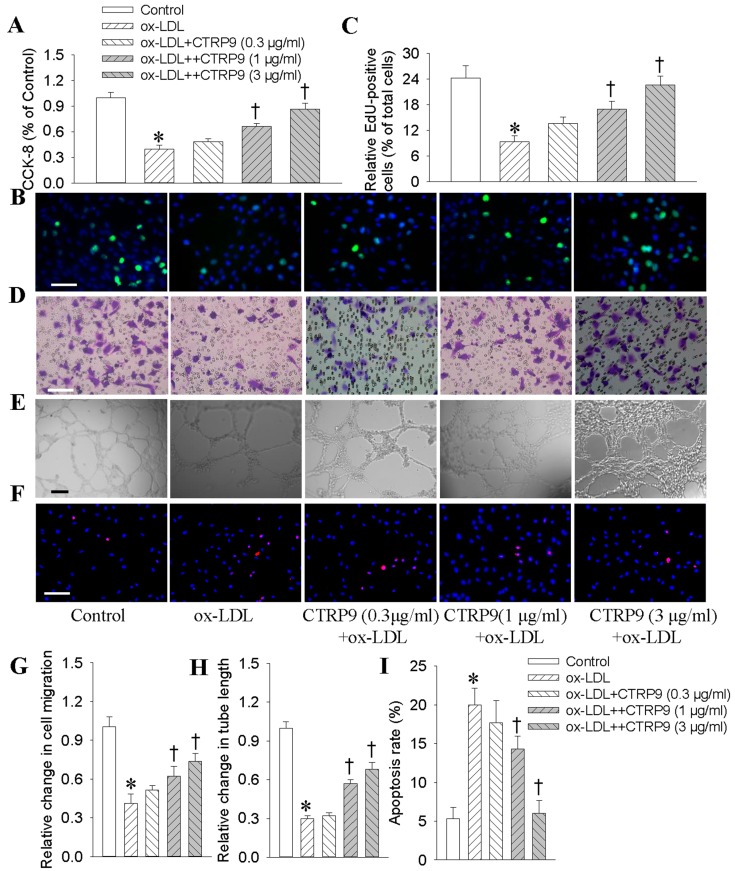
Effects of C1q/TNF-related protein 9 (CTRP9) on the proliferation, migration, angiogenesis, apoptosis in Oxidized low-density lipoprotein-treated (ox-LDL-treated) human umbilical vein endothelial cells (HUVECs). HUVECs were pretreated with different doses of CTRP9 (0.3, 1 and 3 μg/mL) for 6 h before ox-LDL (100 μg/mL) incubation for another 24 h. (**A**) The cell viability was determined by CCK-8 (Cell Counting Kit-8) test; (**B**) Representative photographs showing the DNA synthesis in HUVECs determined with EdU incorporation assay. Scale bar, 100 μm; (**C**) Quantitative analysis of EdU-positive cells by calculating a ratio of EdU (5-ethynyl-2'-deoxyuridine)-positive cells to total cells. Blue fluorescence (Hoechst 33342) shows cell nuclei and green fluorescence (Edu) stands for cells with DNA synthesis. Scale bar, 100 μm; (**D**,**G**) Transwell assays were performed to determine the migration of HUVECs. Scale bar, 100 μm; (**E**,**H**) Matrigel angiogenesis assay in HUVECs. Scale bar, 200 μm; (**F**,**I**) TUNEL-positive nuclei in red fluorescent color and total nuclei staining with DAPI. Scale bar, 100 μm. Values are mean ± SE. * *p* < 0.05 vs. Control, † *p* < 0.05 vs. ox-LDL. *n* = 6 for each group.

CCK-8 (Cell Counting Kit-8) assay showed that the reduced proliferation of human umbilical vein endothelial cells (HUVECs) in response to ox-LDL (100 μg/mL for 24 h) was significantly reversed by CTRP9 (Figure 1A). Meanwhile, no significant cytotoxicity was observed in CTRP9 treatment at given doses (Figure S1). Pretreatment with CTRP9 dose-dependently increased EdU (5-ethynyl-2′-deoxyuridine)-positive HUVECs (Figure 1B,C). In addition, CTRP9 promoted the cell cycle progression as evidenced by reductions in cell populations of G0/G1 phases, and increases in cell populations of S phase (Figure S2). Incubation of HUVECs with CTRP9 accelerated the migration (Figure 1D,G) and tube formation of HUVECs (Figure 1E,H). The apoptosis of HUVECs upon to ox-LDL was obviously prevented by CTRP9 (Figure 1F,I).

### 2.2. CTRP9 Alleviated Oxidative Stress in ox-LDL-Treated HUVECs

**Figure 2:**
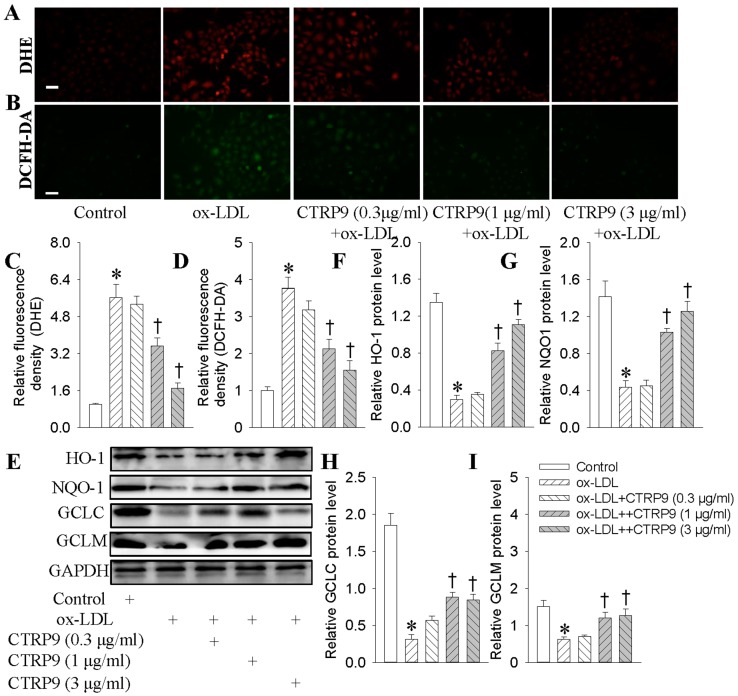
Effects of CTRP9 on the oxidative stress and antioxidant enzymes expressions in ox-LDL-treated HUVECs. HUVECs were pretreated with different doses of CTRP9 (0.3, 1 and 3 μg/mL) for 6 h, and then were exposed to ox-LDL (100 μg/mL) for 24 h; (**A**,**C**) The levels of superoxide anions detected by dihydroethidium (DHE) staining. Scale bar, 100 μm; (**B**,**D**) The ROS levels measured by 2’,7’-dichlorofluorescein diacetate (DCFH-DA). Scale bar, 100 μm; (**E**,**F**) The HO-1 (heme oxygenase-1) protein detected by Western blot; (**E**,**G**) The NQO-1 (NAD(P)H dehydrogenase quinone 1) protein detected by Western blot; (**E**,**H**) The GCLC (glutamate-cysteine ligase catalytic subunit) protein detected by Western blot. (**E**,**I**) The GCLM (glutamate-cysteine ligase modifier subunit) protein detected by Western blot. Values are mean ± SE. * *p* < 0.05 vs. Control, † *p* < 0.05 vs. ox-LDL. *n* = 4 for each group.

HUVECs exhibited increased ROS accumulation, and decreased the levels of antioxidant enzymes including HO-1, NQO-1, GCLC, and GCLM after ox-LDL stimulation (Figure 2). dihydroethidium (DHE) (Figure 2A,C) or 2′,7′-dichlorofluorescein diacetate (DCFH-DA) (Figure 2B,D) fluorescent dye showed that the productions of superoxide anions were increased in ox-LDL-stimulated HUVECs, which were abrogated by CTRP9. Pretreatment with CTRP9 partially reversed ox-LDL-induced reductions of HO-1 (Figure 2E,F), NQO-1 (Figure 2E,G), GCLC (Figure 2E,H), and GCLM (Figure 2E,I) protein expressions. 

### 2.3. Effect of CTRP9 on Nitric Oxide (NO) Generation and Endothelial Nitric Oxide Synthase (eNOS) Levels

**Figure 3:**
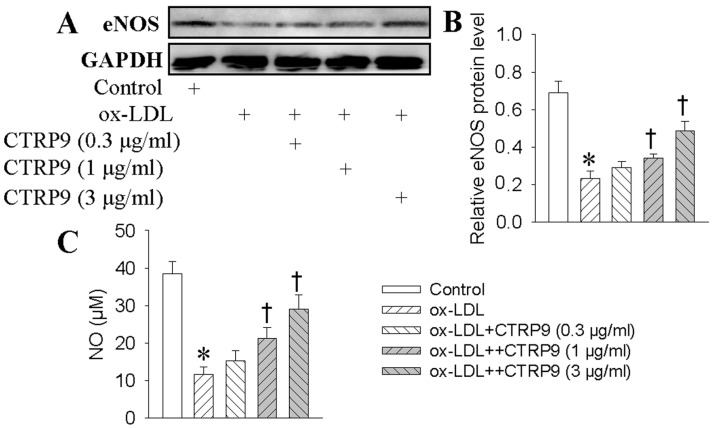
Effects of CTRP9 on the Endothelial Nitric Oxide Synthase (eNOS) expressions and Nitric Oxide (NO) levels in HUVECs stimulated by ox-LDL. HUVECs were pretreated with different doses of CTRP9 (0.3, 1 and 3 μg/mL) for 6 h, and then were exposed to ox-LDL (100 μg/mL) for 24 h. (**A**) Representative images of eNOS protein levels; (**B**) Expression levels are represented as density of eNOS relative to that of glyceraldehyde phosphate dehydrogenase (GAPDH). The level of GAPDH was used as a loading control. Image J software was used to quantify the density of the bands. (**C**) NO levels were quantified by Griess assay. Values are mean ± SE. * *p* < 0.05 vs. Control, † *p* < 0.05 vs. ox-LDL. *n* = 4 for each group.

In the presence of ox-LDL, the protein levels of eNOS were remarkably suppressed, which were largely restored by CTRP9 (Figure 3A,B). The generation of NO in HUVECs was decreased in response to ox-LDL, while the production of NO can be significantly increased by CTRP9 pretreatment (Figure 3C). 

### 2.4. Activation of Adenosine Monophosphate-Activated Protein Kinase (AMPK) Mediated the Protective Effects of CTRP9

**Figure 4:**
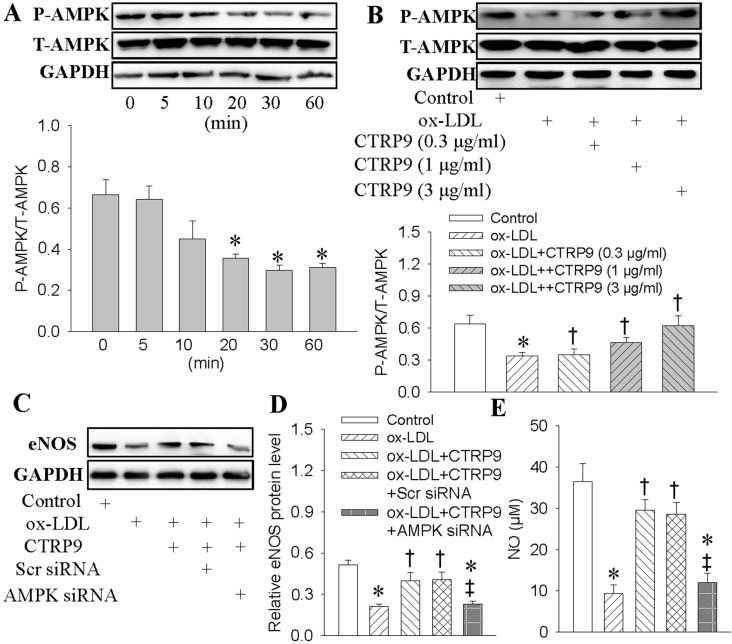
Activation of adenosine monophosphate-activated protein kinase (AMPK) mediated the protective effects of CTRP9 against ox-LDL-evoked injury in HUVECs. (**A**) HUVECs were treated with ox-LDL (100 μg/mL) for different time. The phosphorylated AMPK were determined with Western blot; (**B**) HUVECs were pretreated with CTRP9 (0.3, 1, 3 μg/mL) for 6 h, and then were exposed to ox-LDL (100 μg/mL) for 30 min. The phosphorylated AMPK were determined with Western blot; (**C**,**D**) HUVECs were transfected with Scrambled (Scr) siRNA or AMPK siRNA for 24 h, and then incubated with CTRP9 (3 μg/mL) for 6 h followed by ox-LDL (100 μg/mL) challenge for 30 min. The eNOS protein levels were determined with Western blot; (**E**) NO levels were quantified by Griess assay. Values are mean ± SE. * *p* < 0.05 vs. 0 min, Control, † *p* < 0.05 vs. ox-LDL, ‡ *p* < 0.05 vs. ox-LDL+ CTRP9 or ox-LDL+ CTRP9+Scr siRNA. *n* = 4 for each group from A to D, *n* = 6 for group E.

Incubation of HUVECs with ox-LDL abated the phosphorylated AMPK, reaching its maximal effects at 30 min after ox-LDL treatment (Figure 4A). However, ox-LDL-mediated suppression of phosphorylated AMPK was abrogated by CTRP9 pretreatment (Figure 4B). Immunofluorescence results further confirmed that the downregulated phosphorylated AMPK upon exposure to ox-LDL was obviously rescued by CTRP9 (Figure S3), suggesting a critical role of CTRP9 in the activation of AMPK. Next, we knocked down AMPK gene by transient transfection of specific siRNA, inhibition of AMPK by siRNA ablated the effects of CTRP9 on the eNOS (Figure 4C,D), NO production (Figure 4E), and endothelial dysfunction (Figure S4) in ox-LDL-stimulated HUVECs. 

### 2.5. Proliferator-Activated Receptor γ Co-Activator 1α (PGC1-α) Was Involved in the Protective Effects of CTRP9

**Figure 5:**
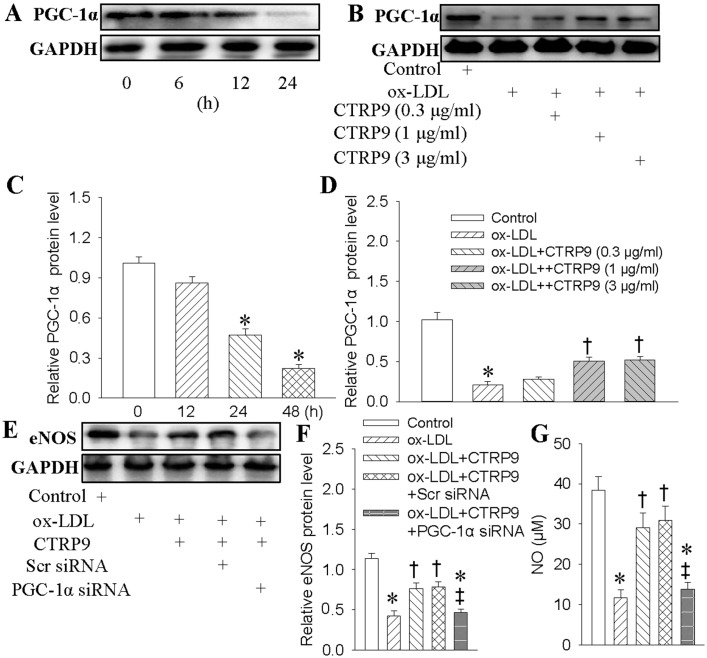
Proliferator-activated receptor γ co-activator 1α (PGC1-α) was involved the protective effects of CTRP9 against ox-LDL-evoked injury in HUVECs. (**A**,**C**) HUVECs were treated with ox-LDL (100 μg/mL) for different time. The PGC1-α protein expression was determined with Western blot; (**B**,**D**) HUVECs were pretreated with CTRP9 (0.3, 1, 3 μg/mL) for 6 h, and then were exposed to ox-LDL (100 μg/mL) for 24 h. The PGC1-α protein expression was determined with Western blot. (**E**,**F**) HUVECs were transfected with Scrambled (Scr) siRNA or PGC-1α siRNA for 24 h, and then incubated with CTRP9 (3 μg/mL) for 6 h followed by ox-LDL (100 μg/mL) challenge for 24 h. The eNOS protein expression was determined with Western blot; (**G**) NO levels were quantified by Griess assay. Values are mean ± SE. * *p* < 0.05 vs. 0 h, Control, † *p* < 0.05 vs. ox-LDL, ‡ *p* < 0.05 vs. ox-LDL+ CTRP9 or ox-LDL+ CTRP9 + Scr siRNA. *n* = 4 for each group from **A** to **F**, *n* = 6 for group G.

In a time-dependent manner, ox-LDL significantly impeded PGC1-α expressions by about 70% of protein at 24 h after stimulation (Figure 5A,C). Nevertheless, the decreased PGC1-α expression was significantly reversed by CTRP9 pretreatment (Figure 5B,D). The siRNA-mediated silencing of PGC1-α eliminated the protective effects of CTRP9 on eNOS (Figure 5E,F), NO production (Figure 5G), and endothelial injury of HUVECs in the context of ox-LDL (Figure S5).

### 2.6. Both AMPK and PGC1-α Participated in CTRP9-Upregulated Antioxidant Enzymes Inductions

**Figure 6:**
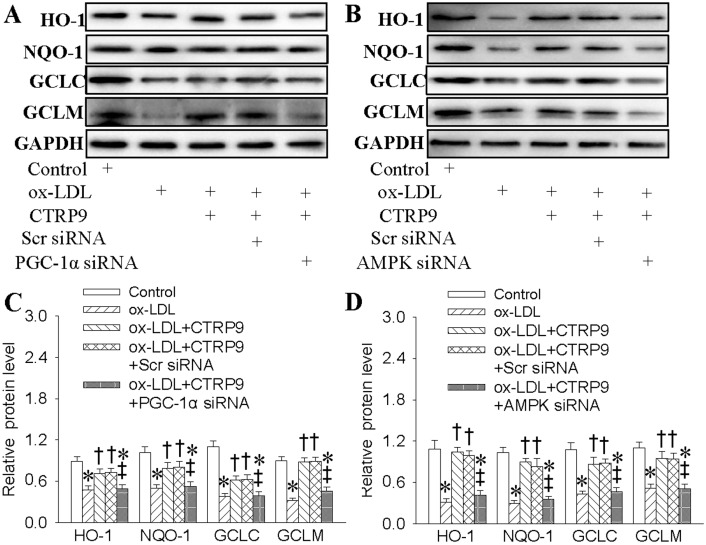
Both AMPK and PGC1-α participated in CTRP9-upregulated antioxidant enzymes in the context of ox-LDL. HUVECs were transfected with Scrambled (Scr) siRNA, PGC-1α siRNA or AMPK siRNA for 24 h, and then incubated with CTRP9 (3 μg/mL) for 6 h followed by ox-LDL (100 μg/mL) challenge for 24 h. (**A**,**C**) Effects of PGC-1α siRNA on the antioxidant enzymes expressions in HUVECs response to ox-LDL. (**B**,**D**) Effects of AMPK siRNA on the antioxidant enzymes expressions in HUVECs response to ox-LDL. Values are mean ± SE. * *p* < 0.05 vs. 0 h, Control, † *p* < 0.05 vs. ox-LDL, ‡ *p* < 0.05 vs. ox-LDL + CTRP9 or ox-LDL+ CTRP9 + Scr siRNA. *n* = 4 for each group.

Both AMPK and PGC1-α are necessary for the protective actions of CTRP9 against ox-LDL-induced oxidative stress. Thus, we examined that whether AMPK and PGC1-α participated in CTRP9-upregulated antioxidant enzymes inductions. As expected, we showed that knockdown of PGC1-α (Figure 6A,C) or inhibition of AMPK (Figure 6B,D) counteracted CTRP9-mediated antioxidant enzymes inductions including HO-1, NQO-1, GCLC, and GCLM, indicating that antioxidant-dependent mechanisms were involved in the beneficial effects of CTRP9 against endothelial dysfunction with regard to ox-LDL stimulation.

## 3. Discussion

The vascular lesions play a crucial role in diabetic, hypertensive, and atherosclerotic complications [4]. The vascular lesions are characterized by platelet activation, coagulation abnormality, fibrinolysis, and dysregulation of endothelial cells, of which endothelial dysfunction is taken as an initial factor for the development of arteriosclerosis [18]. Therapeutic strategies against endothelial dysfunction are promising approaches for the prevention and treatment of vascular lesions [19]. CTRPs belong to one member of the C1q/TNF protein superfamily with beneficial effects on glucose metabolism [15] and vascular function [12]. In the present study, our results demonstrated that CTRP9 played a pivotal role to ameliorate endothelial dysfunction induced by ox-LDL via antioxidant enzymes and eNOS inductions associated with PGC-1α/AMPK activation.

The proliferation, migration, and formation of vessel-like tube structures of endothelial cells coordinately contribute to angiogenesis, which is an essential factor in tissue repair [20]. The angiogenesis of endothelial cells was disrupted by ox-LDL deposition during the development of atherosclerosis [21]. Therefore, repair of damaged endothelial cells or enhancement of new collateral vessel growth may be beneficial for the treatment of various vascular diseases [11]. In the present study, we showed that ox-LDL restrained the proliferation, migration, and angiogenesis of HUVECs, which were supported by many previous studies [22,23,24]. However, CTRP9 reversed ox-LDL-mediated decreases in the proliferation, migration, and angiogenesis of HUVECs. These results suggested that CTRP9 participated in the regulation of angiogenesis.

The endothelial apoptosis in response to ox-LDL promotes the lipids deposition, vascular smooth muscle cell migration, foam cell formation, and the development of atherosclerotic plaque [18]. Administration of CTRP9 attenuated cardiomyocyte apoptosis and myocardial remodeling in mice after acute myocardial infarction [25]. CTRP9 attenuates the apoptosis of primary pulmonary artery epithelial cells, which is essential for amelioration of pulmonary arterial hypertension [26]. In the presence of CTRP9, the cardiac infarction, and cardiomyocytes apoptosis are markedly reversed in myocardial ischemia reperfusion rats [27]. In this present study, we demonstrated that CTRP9 significantly restored the apoptosis of HUVECs in response to ox-LDL. These results hinted that CTRP9 may be employed for endothelial repair via inhibition of endothelial cell apoptosis. 

Vascular endothelium releases a variety of vasoactive substances to regulate endothelial function. Among these molecules, NO is a crucial molecule that promotes the angiogenesis of endothelial cell, vasodilation, and impedes leukocyte adhesion [28]. The impaired NO bioactivity is critically involved in endothelial dysfunction [29]. NO is mainly produced by endothelial eNOS in endothelial cells under physiological condition [30]. In this study, our results displayed that ox-LDL decreased the expression of eNOS and the generation of NO, which was rescued by CTRP9 pretreatment. These results implied that CTRP9 exhibited protective roles in endothelial dysfunction induced by ox-LDL. 

AMPK, an energy sensor, is identified to be ubiquitously expressed in vascular cells, and exerts anti-atherosclerotic effects [31]. The protective effects of adiponectin on cardiovascular diseases are expected to be partially mediated by the activation of AMPK signaling [32]. Metformin improves diabetic endothelial function in association with AMPK activation [33]. AMPK/eNOS pathway is responsible for the protective role of ciglitazone in ox-LDL-induced endothelial cells [34]. AMPK activation is responsible for the anti-inflammatory effect of CTRP9 on endothelial cells [17]. Globular CTRP9 alleviates ox-LDL-evoked inflammation in RAW 264.7 macrophages through AMPK activation [35]. Adenovirus-mediated overexpression of CTRP9 activates AMPK to lower serum glucose levels in obese (ob/ob) mice [15]. Herein, we showed that CTRP9 prevented the ox-LDL-mediated the suppression of AMPK phosphorylation. More importantly, knockdown of AMPK eradicated the inhibitory effects of CTRP9 on endothelial dysfunction in response to ox-LDL, implying the essential role of AMPK. 

PGC-1α is identified as a transcriptional coactivator, and it is a fundamental regulator of mitochondrial function [36]. Various studies highlight an important protective role for PGC-1α in endothelial dysfunction [37]. Overexpression of PGC-1α suppressed tumor necrosis factor-α-induced endothelial inflammation and ROS accumulation in human aortic endothelial cells [38]. Salidroside is demonstrated to protect endothelium dysfunction against H2O2-induced injury via upregulation of PGC-1α [39]. PGC-1α orchestrates cellular defenses against ROS production [36]. Treatment of CTPR9 counteracts high glucose-induced endothelial oxidative damage associated with upregulation of PGC-1α [40]. In the present study, we unveiled that CTRP9 restored the PGC-1α expressions in ox-LDL-treated HUVECs. Furthermore, PGC-1α inhibition abolished CTRP9-mediated endothelial protective function. These results disclosed that CTRP9 may attenuate ox-LDL-stimulated endothelial dysfunction through activation of PGC-1α.

Oxidative stress is also involved in endothelial dysfunction by destruction of NO synthesis [41]. Ox-LDL-mediated endothelial injury destroys the normal integrity of the endothelium via excessive ROS production [42]. NAD(P)H oxidases are the major source of ROS in vascular cells [43]. Antioxidant enzymes including HO-1, NQO1, GCLC and GCLM are well clarified to reduce ROS production [44]. HO-1 induction is a therapeutic strategy in diabetes-induced endothelial dysfunction [45]. Cinnamaldehyde is suggested to alleviate endothelial dysfunction in response to high glucose by upregulation of NQO1 [46]. Quercetin significantly increases glutamate-cysteine ligase (GCL) catalytic and modifier subunits to abrogate LPS-induced oxidant production [47]. Infusion of CTRP9 blocked the superoxide generation, oxidative signaling, but improved cardiac function of diabetic mice [48]. Our results showed that ox-LDL elicited tremendous ROS, but abated the HO-1, NQO-1, GCLC, and GCLM expressions in HUVECs, and these effects were prevented by CTRP9 pretreatment. It is noted that knockdown of PGC1-α or AMPK suppressed CTRP9-mediated antioxidant enzymes inductions including HO-1, NQO-1, GCLC, and GCLM, these results hinted that PGC1-α/AMPK-dependent antioxidant enzymes induction was responsible for the protective actions of CTRP9 in ox-LDL-mediated endothelial dysfunction. 

Collectively, we showed that CTRP9 stimulated upregulation of antioxidant enzymes and eNOS dependent on PGC-1α/AMPK signaling activation, contributing to protect endothelial dysfunction against ox-LDL. CTRP9 may serve as a candidate for protection of vascular endothelium, thus providing a promising alternative for the treatment of endothelial dysfunction in atherosclerosis. 

## 4. Materials and Methods

### 4.1. Reagents and Chemicals

Human CTRP9 was obtained from Aviscera Bioscience Inc. (Santa Clara, CA, USA). Human umbilical vein endothelial cells (HUVECs) were purchased from American Type Culture Collection (Rockville, MD, USA). Cell counting kit-8 (CCK-8) kits, dhydroethidium (DHE), nitric oxide (NO) assay kits were obtained from Beyotime Institute of Biotechnology (Shanghai, China). Ox-LDL (oxidized low density lipoprotein) was purchased from Beijing Solarbio Life Science Company (Beijing, China). EdU incorporation assay kits were purchased from RiboBio (Guangzhou, China). Cell Meter^TM^ Terminal Deoxynucleotidyl Transferase-Mediated Dutp Nick End Labelling (TUNEL) Apoptosis Assay Kit was obtained from AAT Bioquest (Sunnyvale, CA, USA). Matrigel matrix was purchased from BD System (Franklin Lakes, NJ, USA). RNase A, propidium iodide, DCFH-DA (2′,7′-dichlorofluorescin diacetate) were obtained from Sigma Chemical Co. (St. Louis, MO, USA). Antibodies against HO-1 (heme oxygenase-1), NQO1 (NAD(P)H dehydrogenase, quinone 1), GCLC (glutamate-cysteine ligase catalytic subunit), GCLM (glutamate-cysteine ligase modifier subunit), eNOS (endothelial nitric oxide synthase), PGC-1α (peroxisome proliferator-activated receptor γ co-activator 1α), phosphorylated AMPK (adenosine monophosphate-activated protein kinase) were purchased from Abcam (Cambridge, MA, USA). Antibody against total AMPK was obtained from Cell Signaling Technology (Danvers, MA, USA). Antibodies against GAPDH, and the horseradish peroxidase conjugated secondary antibody were purchased from Vazyme Biotech Co., Ltd (Nanjing, China). Targeted sequences of the siRNA against AMPK, PGC-1α and Scrambled siRNA were synthesized by GenePharma Co. (Shanghai, China).

### 4.2. Cell Culture 

Human umbilical vein endothelial cells (HUVECs) were cultured in RPMI 1640 medium supplemented with 10% fetal bovine serum, 100 units/mL penicillin, and 100 µg/mL streptomycin under a condition at 37 °C in a humidified air containing 5% CO_2_ [49,50]. The growth medium was replaced every 1–2 day and the cells were seeded onto petri dishes or multi-well plates at a ratio of 1 to 3 upon 80% confluency.

### 4.3. Cytotoxicity of CTRP9 

Cytotoxicity of CTRP9 was assessed by using a CCK-8 kit as previous report [29,51]. In brief, HUVECs at 80% confluency were seeded onto 96-well plates (4000 cells/well). CTRP9 was diluted to final concentrations from 0 to 30 μg/mL in the medium. After incubation for 24 h at 37 °C, the absorbance was measured by a microplate reader (SYNERGY H4, BioTek, Winooski, VT, USA) at a wavelength of 490 nm.

### 4.4. Cell Proliferation and Cell Cycle Assay

HUVECs were trypsinized and transferred to 96-well plate at 1 × 10^4^ cells/well. The cells were subjected to serum-deprived medium culture overnight, and then exposed to ox-LDL (100 μg/mL) in the presence or absence of CTRP9 (0.3, 1, 3 μg/mL) for 24 h. Finally, 10 μl of CCK-8 solution was added into each well for 2 h at 37 °C. The absorbance was measured at 450 nm using a microplate reader (SYNERGY H4, BioTek, VT, USA). EdU incorporation was also applied to determine the DNA synthesis of HUVECs using In Vitro Imaging Kit (Guangzhou RiboBio, Guangzhou, China) [52,53]. The collected HUVECs were harvested and fixed in ice-cold 70% ethanol overnight at −20 °C. After three washes in PBS, the cells were resuspended in PBS containing 100 μg/mL RNase A (Sigma, St. Louis, MO, USA) and 50 μg/mL propidium iodide (Sigma, St. Louis, MO, USA) for 30 min at in the dark. Cell cycle analysis was conducted with the aid of a FACScalibur cytometer (Becton Dickinson, San Jose, CA, USA), and the cell cycle phases were analyzed with FlowJo software (ver. 10.0, FlowJo LLC, Ashland, OR, USA). The results were set as the percentage of cells in each cell cycle phase [54].

### 4.5. Migration Assay and Tube Formation Assay

The migration of HUVECs was evaluated by a Boyden chamber assay as previously described [55]. Briefly, HUVECs were placed in the upper chamber of Transwell (5 × 10^4^ cells, 8-μm pore size chamber, Corning). After the indicated treatment, HUVECs were allowed to migrate for 24 h at 37 °C. The non-migrated cells were scraped tightly with a cotton swab. The migrated cells in the lower surface of the filter were stained with 1% crystal violet, and the stained cells were photographed by a digital output camera attached to an inverted phase-contrast microscope. The basement membrane matrix (BD Biosciences, Franklin Lakes, NJ, USA) was placed into the well of 96-well plate, and hardened at 37 °C for 30 min [56]. HUVECs were seeded on each well, and incubated with ox-LDL (100 μg/mL) with or without CTRP9 (0.3, 1, 3 μg/mL) for 24 h. Tube formation was observed using a digital output camera attached to an inverted phase-contrast microscope. Tube formation was evaluated using Image J software (ver. 1.43u, National Institutes of Health, Bethesda, MD, USA).

### 4.6. Apoptosis Detection by Terminal Transferase-Mediated dUTP Nick End-Labeling (TUNEL) Staining

The cell apoptosis was examined by the terminal transferase-mediated dUTP nick end-labeling (TUNEL) assay according to the manufacturer’s suggestions [57]. The fixed cells were stained using fluorescein-conjugated TUNEL, and the cell nuclei were stained with 4′,6-diamidino-2-phenylindole (DAPI). The TUNEL-positive cells were observed a fluorescence microscope (80i, Nikon, Japan). The apoptotic rate was quantified by counting TUNEL positive cells from 6 random fields and was expressed as a percentage of total cells.

### 4.7. Nitric Oxide (NO) Generation Measurement

The NO production was evaluated by Griess reaction as previously described [58,59]. The NO detection kit (Beyotime Biotech Inc., Nanjing, China) was used following the instructions by the manufacturer. The cell culture solutions in each well were collected and a standard curve was produced by using NaNO_2_. Fifty microliters of Griess reagent was added to 50 µL of suspending media. After 15 min, the optical density was read in a microplate reader at 540 nm. Each experiment was performed in six samples. NO data was expressed in µM.

### 4.8. Intracellular Reactive Oxygen Species (ROS) Measurement

The ROS production was assessed by dihydroethidium (DHE) or 2′,7′-dichlorofluorescein diacetate (DCFH-DA) as previous report [60,61]. The stimulated HUVECs were washed with phosphate buffer saline (PBS) for three times. The HUVECs were then fixed and treated with DHE (10 μM) or DCFH-DA (10 μM) 20 min at 37 °C, respectively. The fluorescence signals were captured with a multi-detection microplate reader, and quantified with the IMAGE-PRO PLUS 6.0 (Version 6.0, Media Cybernetics, Bethesda, MD, USA) by using the same parameters. The measured fluorescence values were normalized to the fluorescence in control cells.

### 4.9. Immunofluorescence Assay 

After treatment, HUVECs were fixed in 4% formaldehyde and permeabilized with 0.1% Triton X-100 in PBS for 15 min. The cells were blocked with 10% goat serum for 30 min, and incubated with primary antibody rabbit anti-AMPKα1 (phospho T183) overnight at 4 °C. The staining was incubated with Alexa Fluor® 488-conjugated anti-rabbit secondary antibody for 30 min. Nuclei was stained with DAPI. Immunofluorescence signals were visualized on a fluorescence microscope (80i, Nikon, Japan).

### 4.10. siRNA Transfections

HUVECs were seeded on 60 mm plates to form 30–50% confluence on the day before the transfection. Cells were resuspended in fresh medium without antibiotics and transfected separately with small interference RNA (siRNA) sequences against AMPK (100 nM), PGC-1α (100 nM) or a scrambled siRNA (100 nM) using Lipofectamine (Invitrogen, Carlsbad, CA, USA) according to the manufacture’s protocols. The transfected cells were changed by fresh medium supplemented with 10% serum and antibiotics after 6 hours of transfection. The siRNA sequences targeted AMPKα1 were as follows: sense, 5′-UUAAGGCUUCAUCAUCAAUCAUGGU-3′; antisense, 5′-ACCAUGAUUGAUGAUGAAGCCUUAA-3′. The siRNA sequences targeted PGC-1α were as follows: sense, 5′-GGACAGUGAUUUCAGUAAUTT-3′; antisense, 5′-AUUACUGAAAUCACUGUCCTT-3′. The control siRNA sequences were as follows: sense, 5′-UUCUCCGAACGUGUCACGUTT-3′; antisense, 5′-ACGUGACACGUUCGGAGAATT-3′. The siRNA sequences targeting AMPKα1 and PGC-1α have been demonstrated to exhibit enough efficiency silencing AMPKα1 and PGC-1α, as previouslt reported [34,62,63].

### 4.11. Western Blot

The protein of HUVECs were extracted in RIPA lysis, and equal amount of total proteins electrophoresed, blotted, and then incubated with required primary antibodies at 4 °C overnight. The blots were then incubated with appropriate secondary horseradish peroxidase (HRP)-conjugated antibodies, and the immunoreactive proteins were visualized by enhanced chemiluminescence (Millipore Darmstadt, Germany).

### 4.12. Statistical Analysis

All results were defined as mean ± S.E. Comparisons within two groups were made by Student′s *t* test. Statistical analysis was performed by ANOVA/Dunnet *t*-test for multiple group comparisons. The criterion for statistical significance was set at *p* < 0.05.

## Supplementary Materials

Supplementary materials can be found at www.mdpi.com/1422-0067/18/6/1097/s1.
