# Supplementary material for: C1q/TNF-Related Protein-9 Ameliorates Ox-LDL-Induced Endothelial Dysfunction via PGC-1α/AMPK-Mediated Antioxidant Enzyme Induction
Source: Int J Mol Sci. 2017 May 26;18(6):1097. doi: 10.3390/ijms18061097 (PMC5485929; doi:10.3390/ijms18061097)
Supplement: Supplementary file 1 [file ijms-18-01097-s001.pdf]

C1q/TNF-related protein-9 ameliorates ox-LDL-induced endothelial dysfunction via PGC-1α/AMPK-mediated antioxidant enzyme induction

Haijian Sun, Xuexue Zhu, Yuetao Zhou, Weiwei Cai, Liying Qiu

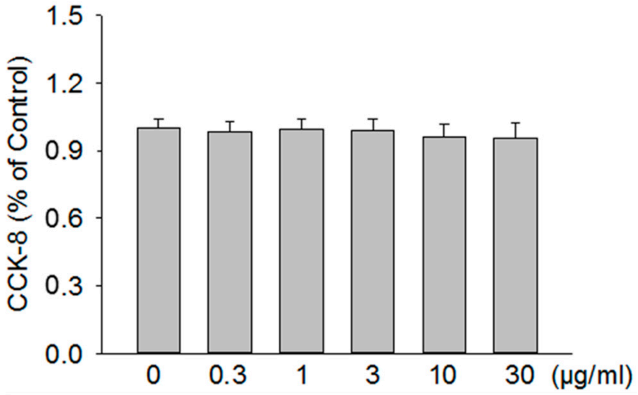

Figure S1. Effect of CTRP9 on the cytotoxicity in HUVECs. HUVECs were treated with CTRP9 at different doses (0, 0.3, 1, 3, 10, 30 μg/mL) for 24 h. Cell viability was measured by CCK-8 assay. Values were expressed as mean ± SE. $n = 6$ for each group.

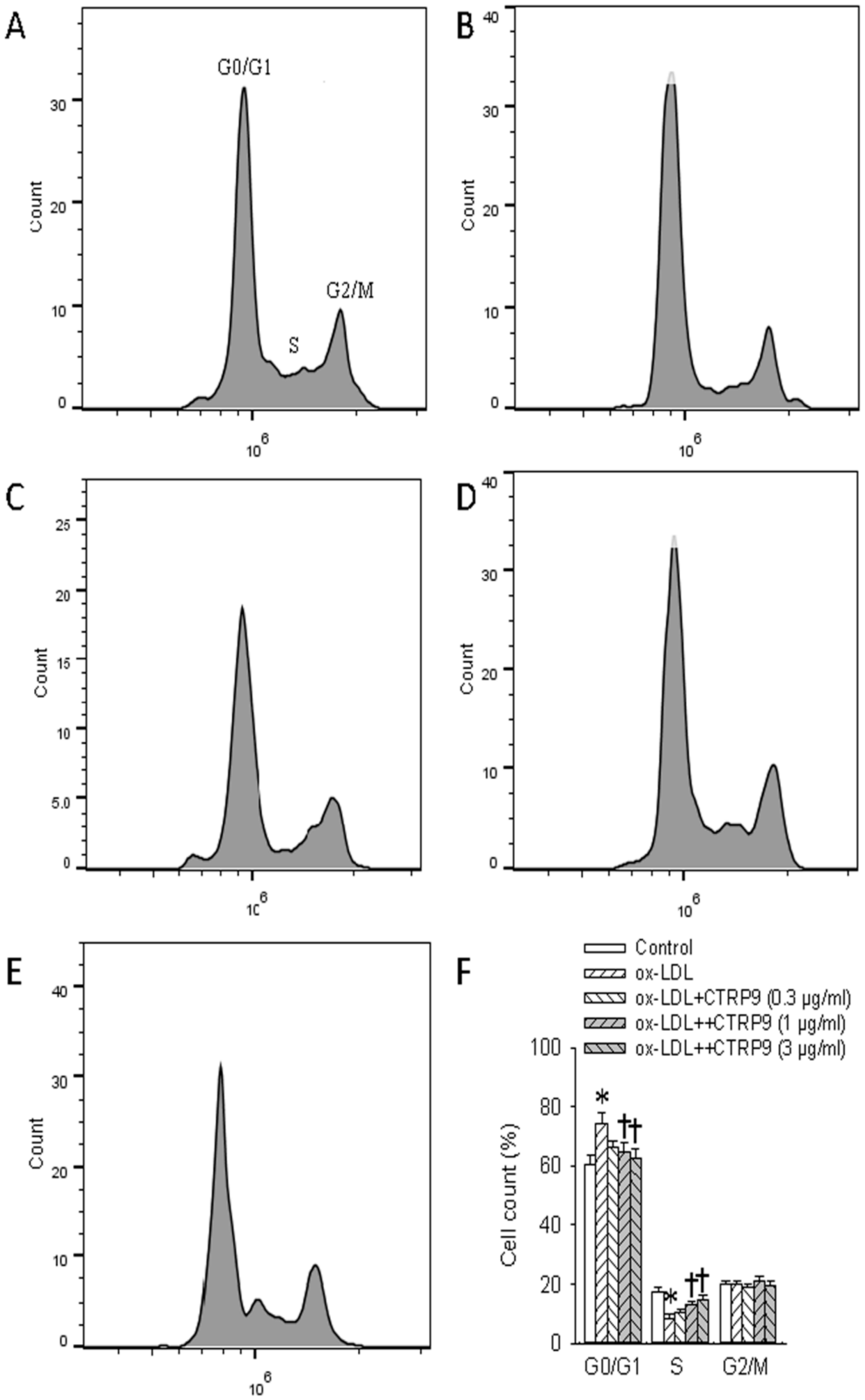

Figure S2. Effects of CTRP9 on the cell cycle in ox-LDL-stimulated HUVECs. HUVECs were pretreated with different doses of CTRP9 (0.1, 1 and 3μg/mL) for 6 h before ox-LDL (100 μg/mL) incubation for another 24 h. (**A**) Control. (**B**) ox-LDL. (**C**) CTRP9 (0.3 μg/mL) + ox-LDL. (**D**) CTRP9 (1 μg/mL) + ox-LDL. (**E**) CTRP9 (3 μg/mL) + ox-LDL. (F) The distribution of various phases in cell cycle evaluated with flow cytometry. Values are mean ± SE. * $P < 0.05$ vs. Control, † $P < 0.05$ vs. ox-LDL. $n = 4$ for each group.

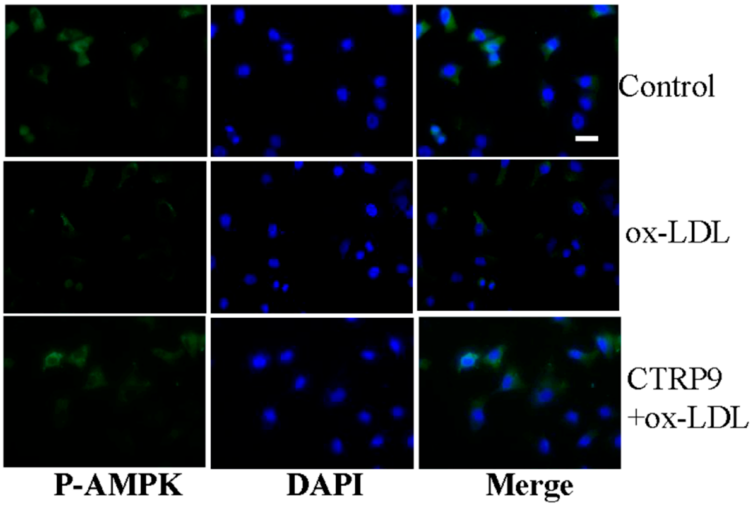

Figure S3. The phosphorylated AMPK was observed by immunofluorescence assay. Scale bar, 50 μm.

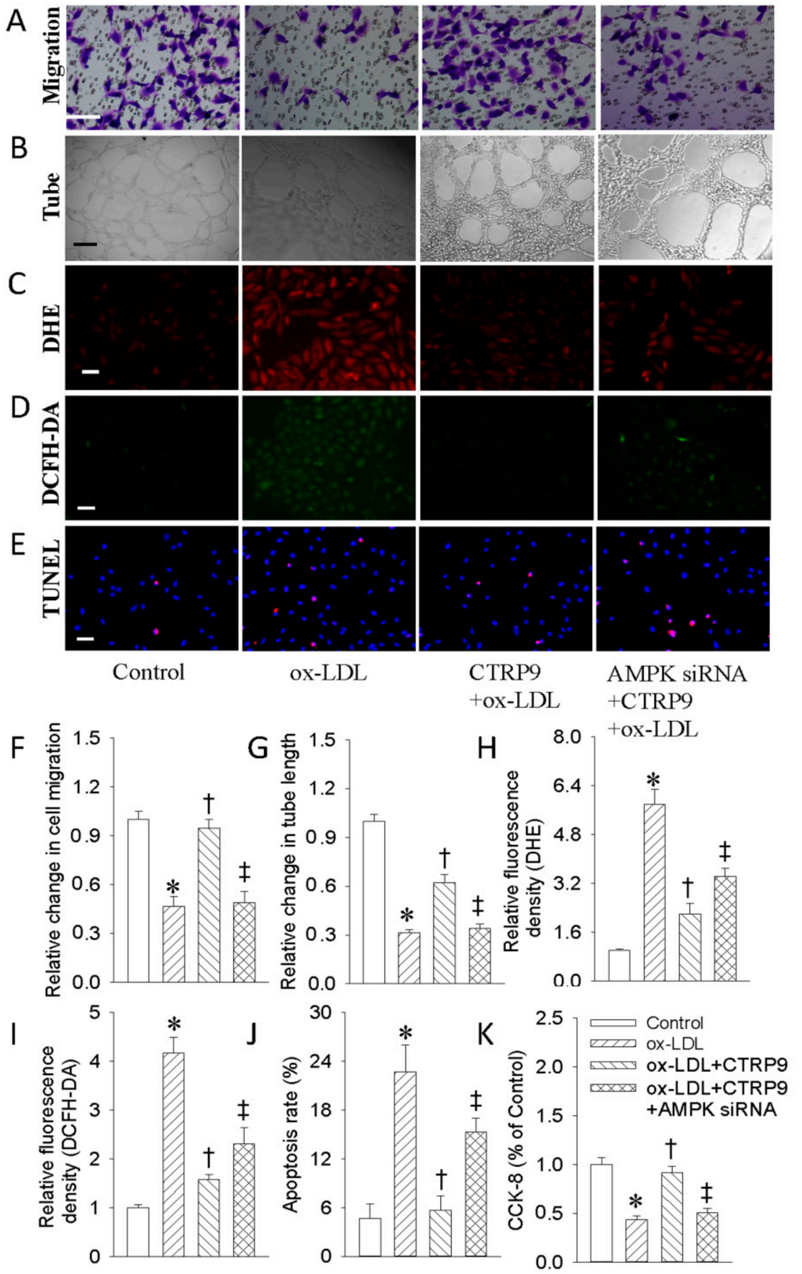

Figure S4. AMPK knockdown abolished antagonistic effects of CTRP9 on the proliferation, migration, angiogenesis, and apoptosis in ox-LDL-treated HUVECs. HUVECs were transfected with AMPK siRNA for 24 h, and then incubated with CTRP9 (3 μg/mL) for 6 h followed by ox-LDL (100 μg/mL) challenge for 24 h. (**A,F**) Transwell assays were performed to determine the migration of HUVECs. Scale bar, 100 μm. (**B,G**) Matrigel angiogenesis assay in HUVECs. Scale bar, 200 μm. (**C,H**) The levels of superoxide anions detected by DHE staining. Scale bar, 100 μm. (**D,I**) The ROS levels measured by DCFH-DA. Scale bar, 100 μm. (**E,J**) TUNEL-positive nuclei in red fluorescent color and total nuclei staining with DAPI. Scale bar, 100 μm. (**K**) The cell viability was determined by CCK-8 test. Values are mean ± SE. * $P < 0.05$ vs. Control, † $P < 0.05$ vs. ox-LDL, ‡ $P < 0.05$ vs. ox-LDL+ CTRP9. $n = 6$ for each group.

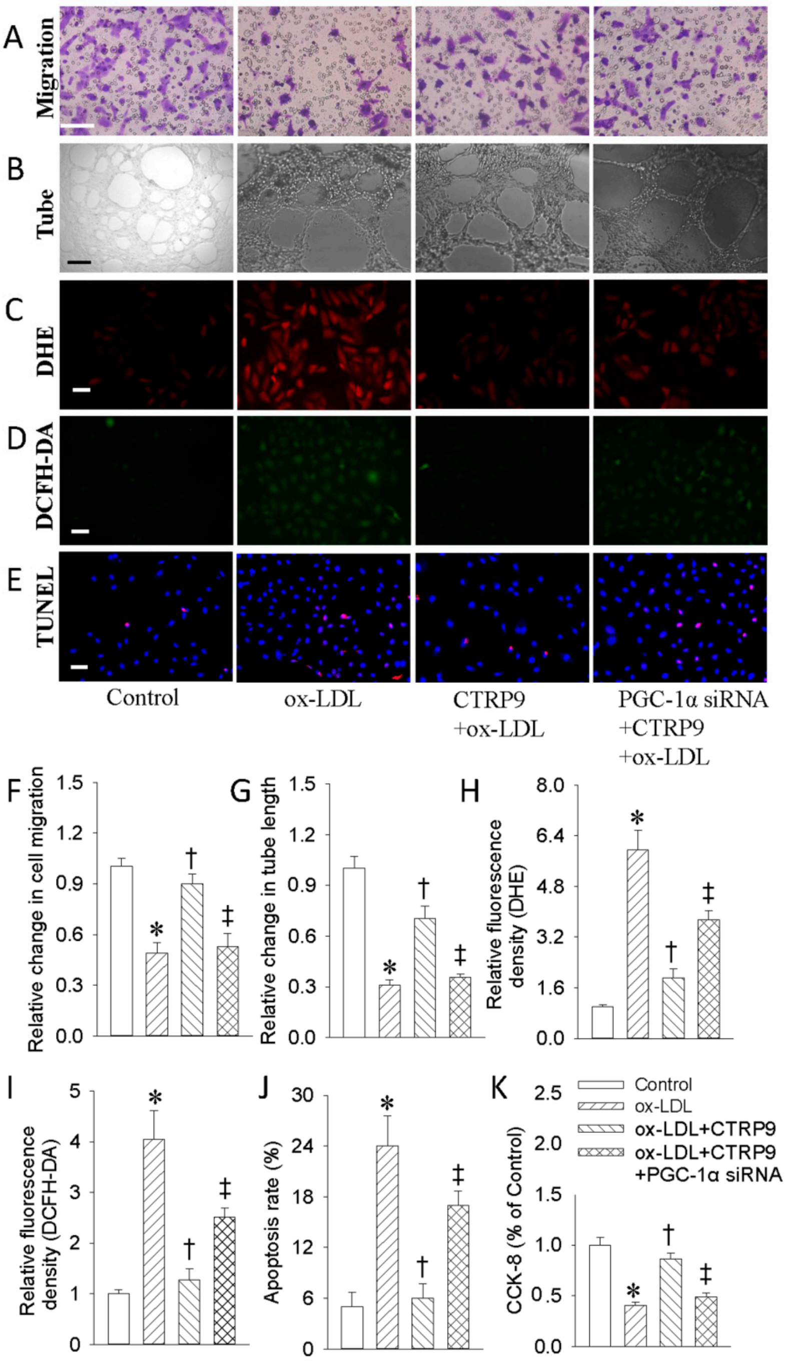

Figure S5. Silencing of PGC1-*α* prevented the protective actions of CTRP9 on the proliferation, migration, angiogenesis, and apoptosis in ox-LDL-treated HUVECs. HUVECs were transfected with PGC1-*α* siRNA for 24 h, and then incubated with CTRP9 (3 μg/mL) for 6 h followed by ox-LDL (100 μg/mL) challenge for 24 h. (**A**,**F**) Transwell assays were performed to determine the migration of HUVECs. Scale bar, 100 μm. (**B**,**G**) Matrigel angiogenesis assay in HUVECs. Scale bar, 200 μm. (**C**,**H**) The levels of superoxide anions detected by DHE staining. Scale bar, 100 μm. (**D**,**I**) The ROS levels measured by DCFH-DA. Scale bar, 100 μm. (**E**,**J**) TUNEL-positive nuclei in red fluorescent color and total nuclei staining with DAPI. Scale bar, 100 μm. (**K**) The cell viability was determined by CCK-8 test. Values are mean ± SE. **P* < 0.05 vs. Control, † *P* < 0.05 vs. ox-LDL, ‡ *P* < 0.05 vs. ox-LDL+ CTRP9. *n* = 6 for each group.
